# IROA: International Register of Open Abdomen, preliminary results

**DOI:** 10.1186/s13017-017-0123-8

**Published:** 2017-02-21

**Authors:** Federico Coccolini, Giulia Montori, Marco Ceresoli, Fausto Catena, Rao Ivatury, Michael Sugrue, Massimo Sartelli, Paola Fugazzola, Davide Corbella, Francesco Salvetti, Ionut Negoi, Monica Zese, Savino Occhionorelli, Stefano Maccatrozzo, Sergei Shlyapnikov, Christian Galatioto, Massimo Chiarugi, Zaza Demetrashvili, Daniele Dondossola, Yovcho Yovtchev, Orestis Ioannidis, Giuseppe Novelli, Mirco Nacoti, Desmond Khor, Kenji Inaba, Demetrios Demetriades, Torsten Kaussen, Asri Che Jusoh, Wagih Ghannam, Boris Sakakushev, Ohad Guetta, Agron Dogjani, Stefano Costa, Sandeep Singh, Dimitrios Damaskos, Arda Isik, Kuo-Ching Yuan, Francesco Trotta, Stefano Rausei, Aleix Martinez-Perez, Giovanni Bellanova, Vinicius Cordeiro Fonseca, Fernando Hernández, Athanasios Marinis, Wellington Fernandes, Martha Quiodettis, Miklosh Bala, Andras Vereczkei, Rafael L. Curado, Gustavo Pereira Fraga, Bruno M. Pereira, Mahir Gachabayov, Guillermo Perez Chagerben, Miguel Leon Arellano, Sefa Ozyazici, Gianluca Costa, Tugan Tezcaner, Luca Ansaloni

**Affiliations:** 1 General, Emergency and Trauma Surgery Department, Papa Giovanni XXIII Hospital, Piazza OMS 1, 24127 Bergamo, Italy; 2 Emergency Surgery Department, Parma University Hospital, Parma, Italy; 3 Virginia Commonwealth University, Richmond, VA USA; 4 General Surgery, Letterkenny Hospital, Donegal, Ireland; 5 General and Emergency Surgery Department, Macerata Hospital, Macerata, Italy; 6 Neuro Intensive Care Unit Department, Papa Giovanni XXIII Hospital, Bergamo, Italy; 7 Emergency Surgery Hospital, Bucharest, Romania; 8 Emergency Surgery Department, Ferrara University Hospital, Ferrara, Italy; 9 Science Research of Emergency Care N. A., Djanelidze, Russia; 10 Azienda Ospedaliera Universitaria Pisana, Pisa, Italy; 11 Kipshidze Central University Hospital, Kipshidze, Georgia; 12 HPB Surgery, Fondazione IRCCS Cà Granda Ospedale Maggiore Policlinico, Milano, Italy; 13 University Hospital “Prof Stoian Kirkovich” AD, Stara Zagora, Bulgaria; 14 Fourth Surgical Department, Hospital George Papanikolau, Aristotle University, Thessaloniki, Greece; 15 General Surgery, Infermi Hospital, Rimini, Italy; 16 Pediatric Intensive Care Unit, Papa Giovanni XXIII Hospital, Bergamo, Italy; 17 LAS + USC Medical Centre, Los Angeles, California USA; 18 Pediatric Intensive Care Unit, Hannover University Hospital, Hannover, Germany; 19 Khuala Krai Hospital, Kuala Krai, Malaysia; 20 Mansoura Faculty of Medicine, Mansoura, Egypt; 21 Medical University of Plovdiv, Plovdiv, Bulgaria; 22 Soroka Medical Centre, Beersheba, Israel; 23 University Hospital of Trauma, Tirana, Albania; 24 Emergency and General Surgery, Fondazione IRCCS Cà Granda Ospedale Maggiore Policlinico, Milano, Italy; 25 Oxford University Hospital, Oxford, UK; 26 John Radcliffe Hospital, Oxford, UK; 27 Erzincan University Faculty of Medicine Mengucek Gazi Training Research Hospital Erzincan, Erzincan, Turkey; 28 Chang Gung Memorial Hospital, Taoyuan, Taiwan; 29 Ospedale Maggiore, Lodi, Italy; 30 Ospedale di Circolo e Fondazione Macchi, University of Insubria, Varese, Italy; 31 Hospital Universitario Doctor Peset, Valencia, Spain; 32 S.S. Annunziata Hospital, Taranto, Italy; 33 Hospital Santa Virgínia, São Paulo, Brazil; 34 Hospital Central Militar, Mexico City, Mexico; 35 Tzaneio General Hospital of Piraeus, Piraeus, Greece; 36 Hospital Regional de Sao Jose, San Jose, Brazil; 37 Hospital Santo Tomás, Panama City, Panama; 38 Hadassah Hebrew University Medical Center, Jerusalem, Israel; 39 Department of Surgery, Medical School University Pécs, Pécs, Hungary; 40 Hospital De Clinicas Da Unicamp, Campinas, Brazil; 41 Vladimir City Clinical Hospital of Emergency Medicine, Vladimir City, Russia; 42 University Hospital, Cuenca, Ecuador; 43 Hospital La Paz, Madrid, Spain; 44 Adana Numune Training and Research Hospital, Department of Surgery, Adana, Turkey; 45 Ospedale Sant’ Andrea University Hospital Sapienza, Rome, Italy; 46 Baskent University School of Medicine, Ankara, Turkey

**Keywords:** Open abdomen, IROA, Register, Peritonitis, Trauma, Ischemia, Vascular emergencies, Compartment, Negative pressure, Commercial, Non-commercial, Bogotà bag, Witmann, Skin, Barker

## Abstract

**Background:**

No definitive data about open abdomen (OA) epidemiology and outcomes exist. The World Society of Emergency Surgery (WSES) and the Panamerican Trauma Society (PTS) promoted the International Register of Open Abdomen (IROA).

**Methods:**

A prospective observational cohort study including patients with an OA treatment. Data were recorded on a web platform (Clinical Registers®) through a dedicated website: www.clinicalregisters.org.

**Results:**

Four hundred two patients enrolled. Adult patients: 369 patients; Mean age: 57.39±18.37; 56% male; Mean BMI: 36±5.6. OA indication: Peritonitis (48.7%), Trauma (20.5%), Vascular Emergencies/Hemorrhage (9.4%), Ischemia (9.1%), Pancreatitis (4.2%),Post-operative abdominal-compartment-syndrome (3.9%), Others (4.2%). The most adopted Temporary-abdominal-closure systems were the commercial negative pressure ones (44.2%). During OA 38% of patients had complications; among them 10.5% had fistula. Definitive closure: 82.8%; Mortality during treatment: 17.2%. Mean duration of OA: 5.39(±4.83) days; Mean number of dressing changes: 0.88(±0.88). After-closure complications: (49.5%) and Mortality: (9%). No significant associations among TACT, indications, mortality, complications and fistula. A linear correlationexists between days of OA and complications (Pearson linear correlation = 0.326 *p*<0.0001) and with the fistula development (Pearson = 0.146 *p*= 0.016).

Pediatric patients: 33 patients. Mean age: 5.91±(3.68) years; 60% male. Mortality: 3.4%; Complications: 44.8%; Fistula: 3.4%. Mean duration of OA: 3.22(±3.09) days.

**Conclusion:**

Temporary abdominal closure is reliable and safe. The different techniques account for different results according to the different indications. In peritonitis commercial negative pressure temporary closure seems to improve results. In trauma skin-closure and Bogotà-bag seem to improve results.

**Trial registration:**

ClinicalTrials.gov NCT02382770

## Background

Temporary abdominal closure technique (TACT) or open abdomen (OA) techniques were firstly described more than 120 years ago [1]. OA procedure is defined as intentionally leaving the fascial edges of the abdomen un-approximated (laparostomy). Since that moment, this technique has been utilized decade by decade more frequently. The “old” paradigm of closing the abdomen at “any cost” has been definitely overcome by the literature evidence. However, no definitive data about OA epidemiology and outcomes exist even if in many cases such as trauma, abdominal sepsis, severe acute pancreatitis, and more in general all those situations in which an intra-abdominal hypertension condition is present and/or when is necessary to prevent the development of abdominal compartment syndrome (ACS), the OA is applied. Moreover, patients treated with OA procedures are absolutely heterogeneous even within the same study and large cohorts of patients treated with the same procedures are rare [2–7]. To overcome this lack of high level of evidence data about the OA indications, management, definitive closure, and follow-up, the World Society of Emergency Surgery (WSES) and the Panamerican Trauma Society (PTS) promoted the International Register of Open Abdomen (IROA) [2].

The present study reports preliminary data from the first 16 months of IROA activity.

## Methods

This is a prospective observational cohort study including patients with an open abdomen treatment. There were no exclusion criteria whereas the only inclusion criterion was the OA treatment. Data were recorded on a web platform (Clinical Registers®) through a dedicated website: www.clinicalregisters.org. Each center inserted data about its patients. Data were recorded according to the study protocol, approved by the coordinating center Ethical Committee (Papa Giovanni XXIII Hospital, Bergamo, Italy) and also registered to ClinicalTrials.gov (ClinicalTrials.gov Identifier: NCT02382770). For each patient, the following were recorded: demographical data, indication to the treatment, TACT, duration of the treatment and number of dressing changes, complications, enteric fistula and mortality before and after closure, according to the study protocol.

All the patients less than 14 years old were considered pediatric and were analyzed separately.

Indications were organized into seven groups (peritonitis, pancreatitis, ischemia, vascular emergencies and hemorrhage, post-operative ACS, trauma and other). TACTs were summarized in four subgroups (Bogotà bag + skin closure, Barker vacuum pack, negative pressure wound therapy (NPWT) assisted and Wittmann patch) to allow an appropriate number of patients in each group.

### Statistical analysis

Continuous variables were expressed as mean and standard deviation and were compared with the ANOVA test; categorical data were expressed as proportions and were compared with the chi square test. Linear associations were tested with the Pearson’s linear correlation model. Data about mortality, definitive closure, and number of days with open abdomen were graphically plotted with the Kaplan-Meyer method for the different techniques and indications (patients who died during treatment were considered as never closed with a length of treatment = ∞).

All the statistical analyses were performed with IBM SPSS 20 (IBM Corp. Released 2011. IBM SPSS Statistics for Windows, Version 20.0. Armonk, NY: IBM Corp.).

## Results

**Fig. 1:**
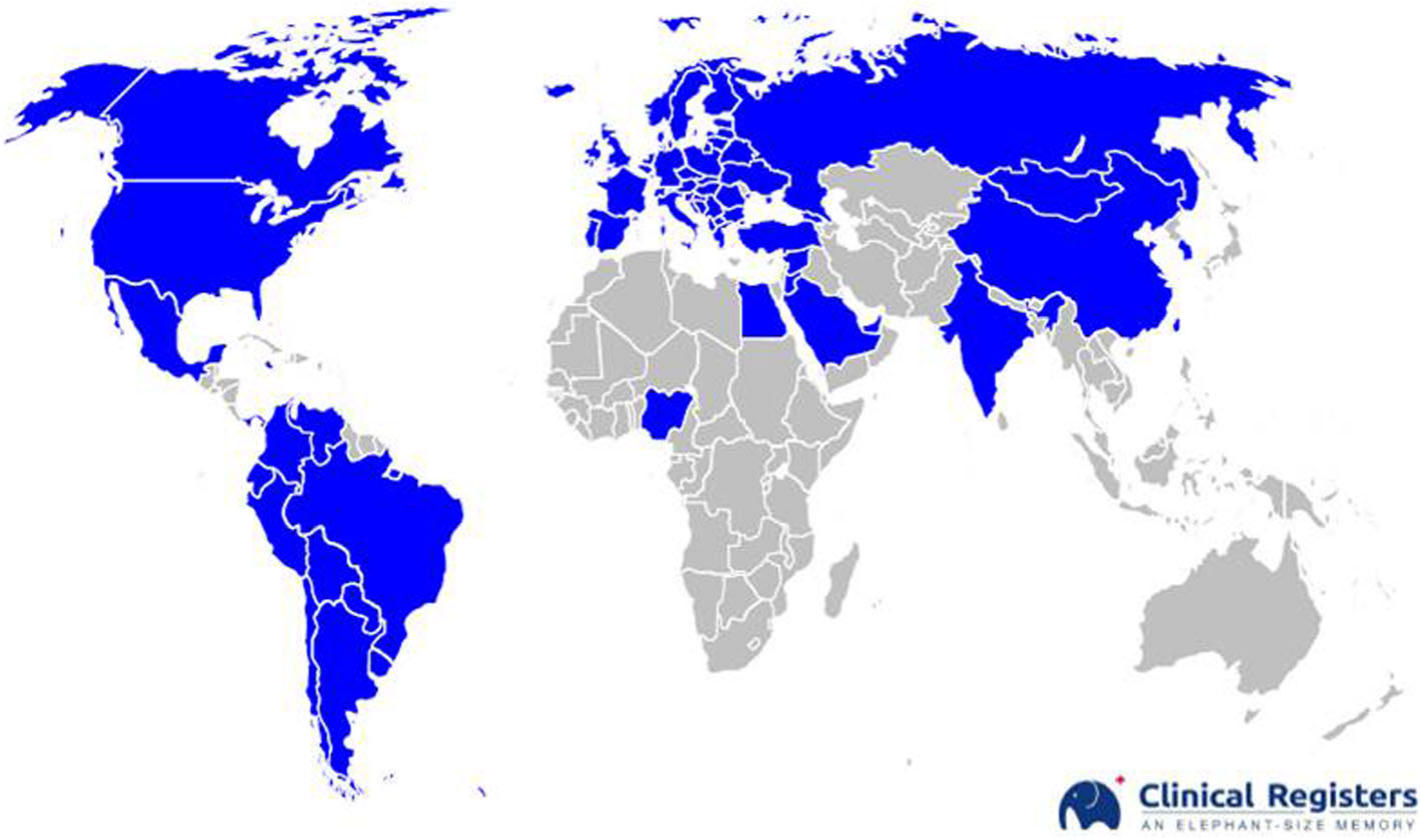
IROA spread in the world

From May 1, 2015, to September 30, 2016, a total number of 402 patients were enrolled and recorded into the register. The IROA spread throughout the world as shown in Fig. 1.

### Adult patients

**Fig. 2:**
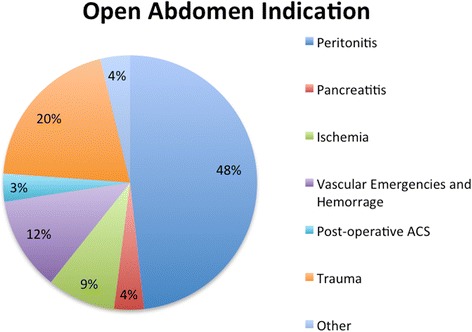
Open abdomen treatment indications

A total of 369 adult patients were recorded; mean age was 57.39 ± 18.37 and 56% were male. The most frequent indication for open abdomen was peritonitis (48.7%) and the most adopted TACT was the commercial negative pressure wound therapy system (44.2%) (Fig. 2, Table 1). During the open treatment, 38% of patients had complications and 10.5% developed an enteric fistula. Definitive closure was achieved in 82.8% of the patients with a mortality during treatment of 17.2%. The mean duration of the open treatment was 5.39(±4.83) days with a mean number of dressing changes of 0.88(±0.88). After-closure complications were recorded in 49.5% of the patients and mortality was 9%.

**Table 1:** Outcomes divided for open abdomen treatment technique (TAC: temporary abdominal closure, NPWT: negative pressure wound therapy)

TAC technique	No. of patients [%] (total = 369)	Age [mean(SD)]	Male gender [%]	No. of dressing [*n*(SD)]	Days of open abdomen [*n*(SD)]	Definitive closure [%]	Fascia closure [%]	Complications during treatment [%]	Fistula [%]	Mortality during open [%]
Bogotà bag + skin closure	31.8	56.5 (18.9)	52.5	0.7 (1.1)	5.0 (4.4)	83.2	71.3	35.8	7.4	16.8
NPWT assisted	44.2	58.7 (17.9)	56.9	1.0 (1.6)	5.0 (4.1)	85.7	59.9	32.5	13.5	14.3
Barker vacuum pack	12.7	50.1 (19.9)	69.0	1.1 (1.6)	6.6 (7.2)	75.6	64.3	43.9	2.4	24.4
Wittmann patch	11.4	63 (14.8)	45.7	0.3 (0.6)	6.6 (4.8)	79.4	65.7	58.8	17.6	20.6
Total	100.0	57.4 (18.4)	55.8	0.9 (1.4)	5.4 (4.8)	82.8	64.7	38.2	10.5	17.2

Tables 1 and 2 show data in detail divided for indication and TACT. Table 3 shows data in details for peritonitis and trauma patients.

**Table 2:** Outcomes divided for indication to open abdomen treatment

Indication	No. of patients [*n*(%)]	Age [mean(SD)]	Male gender [%]	No. of dressing [*n*(SD)]	Days of open abdomen [*n*(SD)]	Definitive closure [%]	Fascia closure [%]	Complications during treatment [%]	Fistula [%]	Mortality during open [%]
Peritonitis	178 (48.7%)	61.9 (14.6)	49.3	0.9 (1.4)	5.2 (4.0)	79.9	59.3	43.2	14.4	20.1
Pancreatitis	14 (4.2%)	60.9 (15.3)	69.2	2.1 (2.2)	12.1 (7.1)	76.9	53.8	53.8	7.7	23.1
Ischemia	32 (9.1%)	69.8 (11.9)	57.1	0.9 (0.9)	5.7 (3.4)	92.9	78.6	39.3	14.3	7.1
Vascular emergencies and hemorrhage	44 (9.4%)	64.6 (12.6)	41.4	0.4 (0.8)	3.7 (3.4)	88.5	72.4	23.1	0.0	11.5
Post-operative ACS	13 (3.9%)	46.8 (20.1)	25.0	0.4 (0.7)	3.9 (2.6)	66.7	50.0	58.3	8.3	33.3
Trauma	74 (20.5%)	39.5 (18.3)	79.4	0.9 (1.6)	5.4 (6.2)	86.4	73.0	20.3	6.8	13.6
Other	14 (4.2%)	57.3 (19.3)	53.8	1.4 (1.8)	5.9 (5.5)	75.0	46.2	41.7	8.3	25.0

**Table 3:** Outcomes in peritonitis and trauma patients (TAC: temporary abdominal closure, NPWT: negative pressure wound therapy)

Indication	TACT	No. of patients [%] (total = 178)	Male gender [%]	Definitive closure [%]	Fascia closure [%]	Complications during treatment [%]	Fistula [%]	Mortality during open [%]
Peritonitis	Bogotà bag + skin closure	28.7	46.5	72.5	62.8	40.0	12.5	27.5
	NPWT assisted	46.0	49.3	85.7	53.6	33.3	14.3	14.3
	Barker vacuum pack	7.3	63.6	80.0	72.7	70.0	0.0	20.0
	Wittmann patch	18.0	48.1	76.9	63.0	61.5	23.1	23.1
	Total	100.0	49.3	79.9	59.3	43.2	14.4	20.1
Trauma	Bogotà bag + skin closure	49.2	71.0	92.9	74.2	25.0	7.1	7.1
	NPWT assisted	28.6	94.4	88.2	77.8	17.6	11.8	11.8
	Barker vacuum pack	22.2	78.6	71.4	64.3	14.3	0.0	28.6
	Total	100.0	79.4	86.4	73.0	20.3	6.8	13.6

**Fig. 3:**
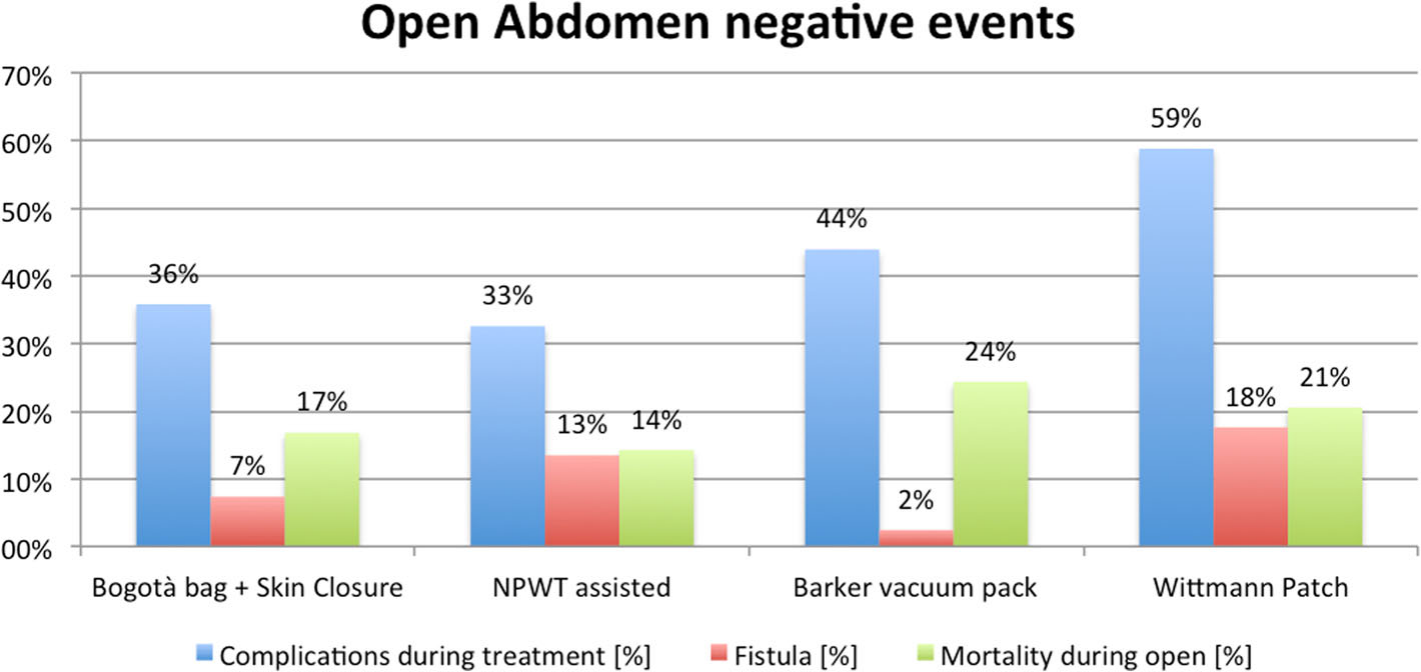
Overall negative event rate (NPWT: negative pressure wound therapy)

**Fig. 4:**
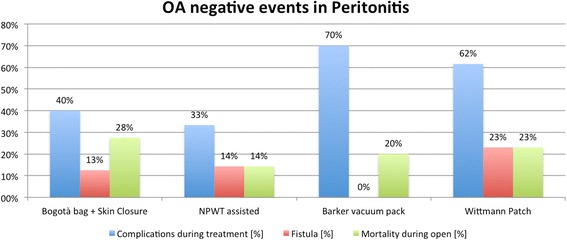
Negative event rate in peritonitis (NPWT: negative pressure wound therapy)

**Fig. 5:**
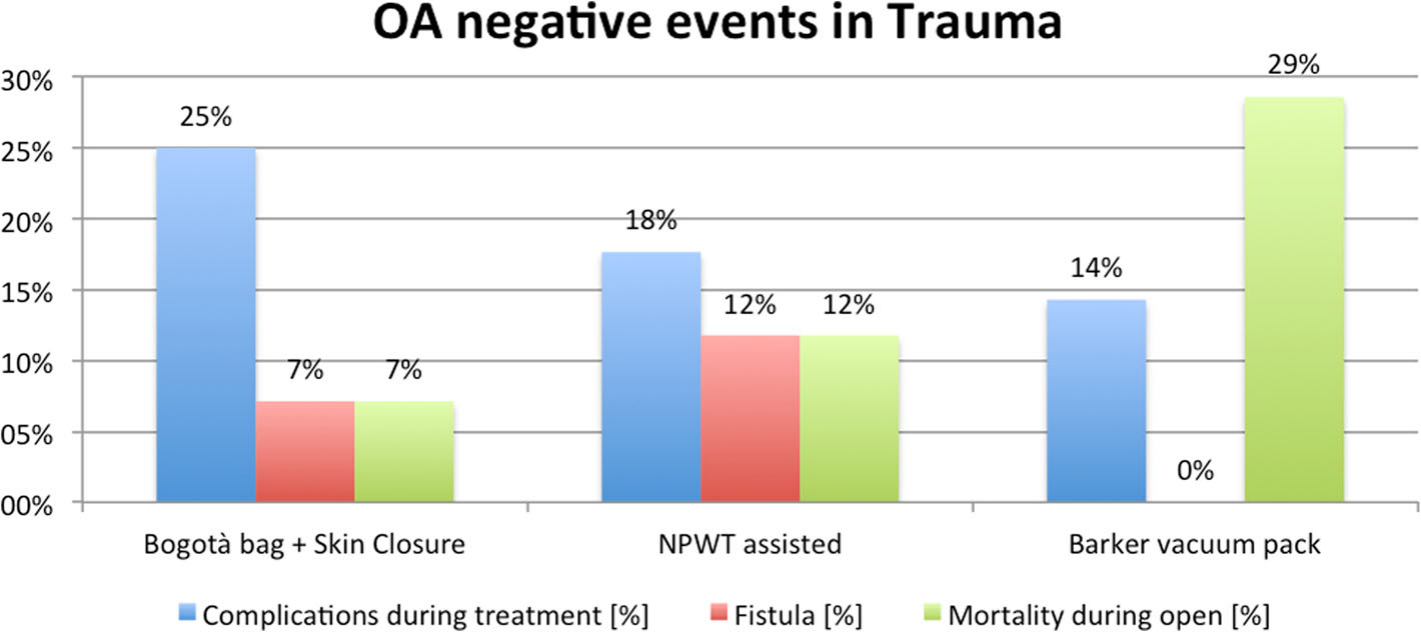
Negative event rate in trauma (NPWT: negative pressure wound therapy)

There were no significant associations among TACT, indications, mortality, complications, and fistula formation. Figures 3, 4, and 5 show the incidence of complications and enteric fistula.

**Fig. 6:**
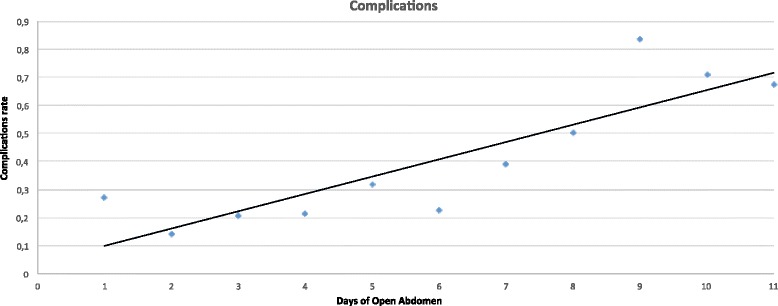
Time distribution of overall complication

**Fig. 7:**
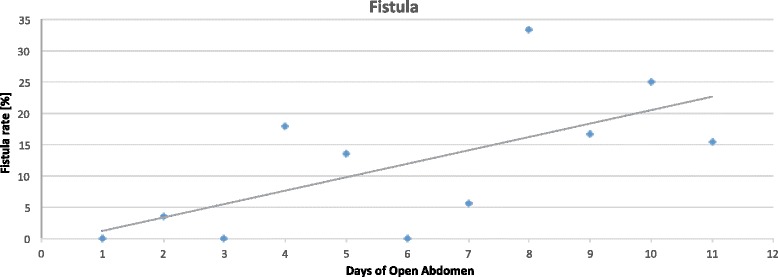
Time distribution of fistula

There was a linear correlation between days of open abdomen and complications (Pearson linear correlation = 0.326 *p* < 0.0001) and with the development of fistula (Pearson = 0.146 *p* = 0.016) (Figs. 6 and 7).

Among the indications, the duration of open treatment was longer for pancreatitis (*p* < 0.0001); no differences were found among different TACTs.

**Fig. 8:**
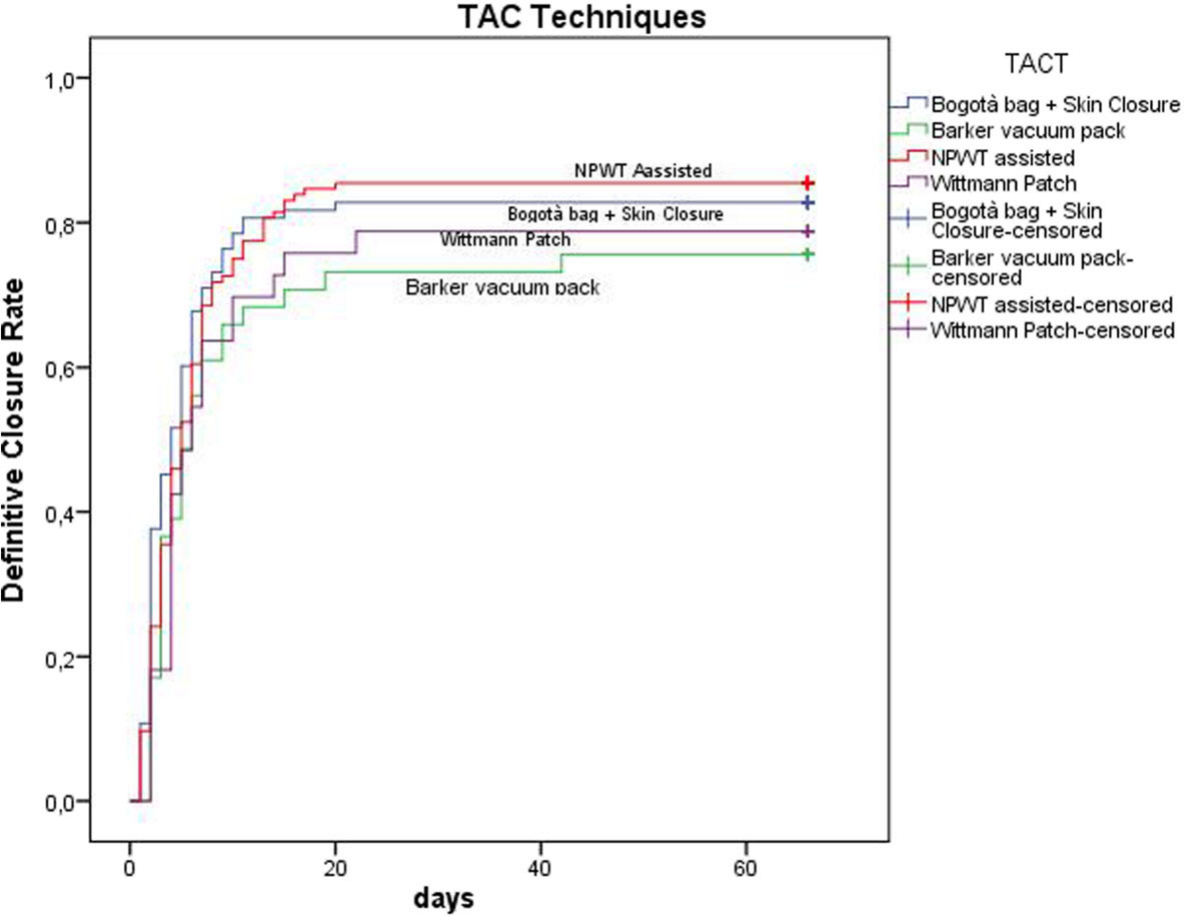
Definitive closure rate and days of open abdomen among different TAC techniques. Those patients died during treatment never achieved definitive closure and had a duration of treatment = infinite (as a consequence asymptotic curve indicates also survival). (TAC: temporary abdominal closure, NPWT: negative pressure wound therapy)

**Fig. 9:**
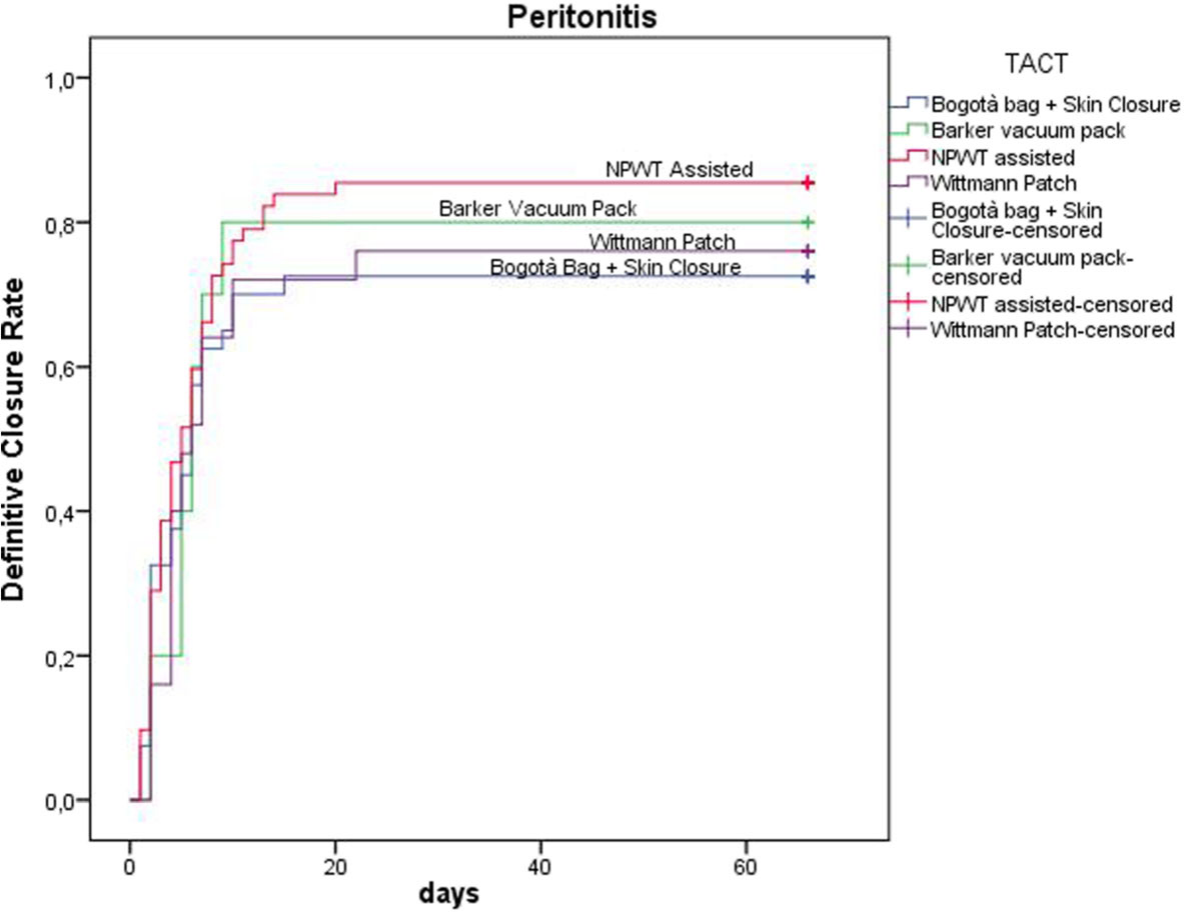
Definitive closure rate and days of open abdomen among different TAC techniques in patients treated for peritonitis. Those patients died during treatment never achieved definitive closure and had a duration of treatment = infinite (as a consequence asymptotic curve indicates also survival). (TAC: temporary abdominal closure, NPWT: negative pressure wound therapy)

**Fig. 10:**
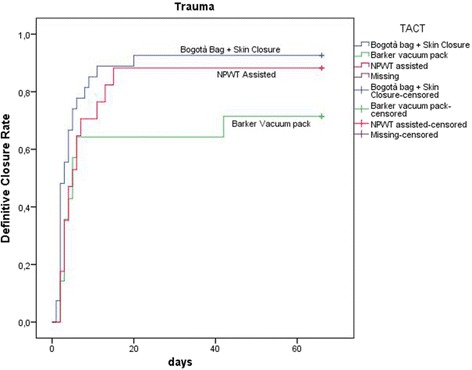
Definitive closure rate and days of open abdomen among different TAC techniques in patients treated for trauma. Those patients died during treatment never achieved definitive closure and had a duration of treatment = infinite (as a consequence asymptotic curve indicates also survival). (TAC: temporary abdominal closure, NPWT: negative pressure wound therapy)

Figures 8, 9, and 10 plotted the days of open abdomen together with the definitive closure rate, shown with the Kaplan-Meyer method, for different TACTs and respectively overall, in peritonitis and in trauma patients.

### Pediatric patients

A total number of 33 pediatric patients were enrolled: mean age was 5.91 ± (3.68) years and 60% were male. Table 4 shows data in detail. The indications for open abdomen were missing for the majority of patients; the most common indicated were various (other), peritonitis, and post-operative ACS. Mortality was recorded in only one patient (3.4%) and complications were recorded in 44.8%; one patient (3.4%) developed enteric fistula. The open treatment had a mean duration of 3.22(±3.09) days.

**Table 4:** Outcomes in pediatric patients (TAC: temporary abdominal closure, NPWT: negative pressure wound therapy)

Open abdomen in pediatrics	
No. of patients	33
Age	5.9 (3.7)
Male gender	60.6%
Indications	
Peritonitis	12%
Pancreatitis	6%
Vascular emergencies and hemorrhage	6%
Post-operative ACS	12%
Other	15%
Missing	48%
No. of dressings	0.3 (0.9)
Days of open abdomen	3.2 (3.1)
Fistula	3.4%
Mortality during treatment	3.4%
Definitive closure	96.6%
Complications post closure	53.6%
Mortality post closure	0.0%
TAC techniques	
Bogotà bag + skin closure	30%
Barker vacuum pack	35%
NPWT assisted	35%

## Discussion

Present data, even if preliminary, explain clearly the worldwide diffusion of such techniques. The most part of cases registered are from adult patients where promising results can be extracted: first of all, the usefulness of OA in acute care in managing severe peritonitis. Acosta et al. in 2011 [8] already described the OA use in Europe demonstrating as more than the 50% of cases of OA were derived from peritonitis patients. Trauma patients represent the second cohort in terms of numerosity. Other interesting results are emerging regarding the different techniques adopted. The most diffused are the commercial negative pressure techniques. As a counterpart, skin closure and Bogotà bag are used in more than 20% of patients with interesting results especially in trauma patients. As recently demonstrated by Kirkpatrick et al., the commercial negative pressure systems obtain better results in term of survival especially in those patients with intra-abdominal infections or contamination [9], or at least in case possibly associated to pro-inflammatory cytokines increased release. The most effective is the removal of infected and cytokine-loaded fluids the better seems to be survival results. Even with no definitive data regarding the effect on the circulating load of cytokines and toxins, maybe the negative pressure will be found to be useful also in reducing these values. Kirkpatrick et al. tried to demonstrate this with their randomized trial in a mixed court of patients [9]. Their results offered one possible way to understand the OA effect related to the utilized TACT. As clearly demonstrated by present data, the different OA techniques are differently useful in each indication. In fact in patients affected by peritonitis the negative pressure systems seem to be the most effective in reducing the mortality rate; moreover, considering the commercial and not commercial systems, the commercial ones seem to be the best in improving survival results (Fig. 9) (see figure legend). As a counterpart in trauma patients, the non-negative pressure systems seem to provide better results if compared to the negative pressure ones (Fig. 10) (see figure legend). This can be partially explained with the relative absence of infection and cytokines to be cleared. Moreover, the closure time is positively influenced by the most appropriate TACT in the different indications (Figs. 9 and 10). In fact closure times differ within the indications and can be partially considered as strictly linked to the utilized TACT.

In terms of complication rate, the different techniques differ one from each other. In absolute, the less the abdomen remains opened the lesser the complication rate. Miller et al. in a big cohort study showed that 8 days of OA represents a cutoff in the complication rate [10]. Present data clearly show that no cutoff can be posed in the complication rate, it progressively increases day by day. The longer the period of OA the higher the number of complications and fistula, starting directly from the very first days of treatment (Figs. 6 and 7). This result is important if analyzed in association with the different outcomes related to the technique of OA and the indications. In fact, analyzing Figs. 8, 9, and 10 (see figure legend), it is possible to see how the two variables are intrinsically connected to the time of closure; the OA technique influences the survival and the time to closure in the different indications, as a counterpart, the indication (i.e., the cause of OA) plays a fundamental role in survival and closure decisions. Both by influencing the time to closure determine part of the causes of the complication insurgence. So it is becoming progressively more evident as it is necessary to reanalyze the TACTs under a different view. The different indications have completely different underlying physiopathology; as a consequence, they maybe are not to be treated with the same TACT. In fact the different techniques possess different characteristic allowing managing different situations. Maybe the application of one technique instead than one-other should not be based only on the availability of the most advanced systems but also on the consideration that each technique has a proper effect on the physiopathology. If these preliminary results will be confirmed by subsequent data, this will lead also to an optimization of the resources located to OA management. Moreover, it would promote future development and researches also regarding the “less technological techniques”.

Within the different indications, the different TACT systems account for different complication rates. As showed in Table 1 and Figs. 3, 4, and 5, the several TACTs seem to lead to different incidence of complication. In evaluating these data however, the underlying cause of OA and the consequent physiopathology conditioning of the outcome should be kept in mind. The technique alone is not completely responsible for all the complications. Some complications such as fistula, however, seem to be more related to the TACT than others.

The incidence of fistula in OA has been reported variously depending on the indication for the OA varying from 4.5 to 25% in trauma [11] and from 5.7 and 17.2% in non-trauma patients [12]. Fistula increase considerably mortality, length of stays, and costs [13]. Present paper confirms data of the literature. The difference in fistula incidence depends also from the adopted TAC technique used. As shown, some techniques account for a higher fistula incidence but as also showed by data different indication have different time of OA and the longer the OA the higher the overall complication and fistula rate. Again, the correlation between TACT, indication, and underlying physiopathology must be kept in mind.

The use of OA in pediatric setting is diffused but not sufficiently studied. Present register is trying to overcome this lack of data. The pediatric data recruitment however must be improved. In fact from the pediatric cohort, no conclusion can be obtained.

One last consideration should be done regarding the limitation of using clinical registries data for evaluating the outcome of rare diseases or those conditions such as emergency interventions in which a randomized controlled trial is rather difficult or even impossible to realize. Results from registries as any non-randomized comparison are affected by the same methodological limitations. Specifically, the comparability of selected patient groups is not ensured. As a counterpart, the only way to obtain wide and trustful results in these situations is the use of dedicated registries; a strong attempt should be done in projecting them to make included patients as much uniform as possible.

## Conclusions

Temporary abdominal closure is reliable and safe in treating severely injured and acute care surgery patients. The different techniques account for different results according to the different indications. In peritonitis, commercial negative pressure temporary closure seems to improve results. In trauma, skin closure and Bogotà bag seem to improve results.

## Abbreviations

ACS: Abdominal compartment syndrome
IROA: International Register of Open Abdomen
NPWT: Negative pressure wound therapy
OA: Open abdomen
PTS: Panamerican Trauma Society
TACT: Temporary abdominal closure technique
WSES: World Society of Emergency Surgery
