# Erratum to: IROA: International Register of Open Abdomen, preliminary results

**DOI:** 10.1186/s13017-017-0127-4

**Published:** 2017-03-09

**Authors:** Federico Coccolini, Giulia Montori, Marco Ceresoli, Fausto Catena, Rao Ivatury, Michael Sugrue, Massimo Sartelli, Paola Fugazzola, Davide Corbella, Francesco Salvetti, Ionut Negoi, Monica Zese, Savino Occhionorelli, Stefano Maccatrozzo, Sergei Shlyapnikov, Christian Galatioto, Massimo Chiarugi, Zaza Demetrashvili, Daniele Dondossola, Yovcho Yovtchev, Orestis Ioannidis, Giuseppe Novelli, Mirco Nacoti, Desmond Khor, Kenji Inaba, Demetrios Demetriades, Torsten Kaussen, Asri Che Jusoh, Wagih Ghannam, Boris Sakakushev, Ohad Guetta, Agron Dogjani, Stefano Costa, Sandeep Singh, Dimitrios Damaskos, Arda Isik, Kuo-Ching Yuan, Francesco Trotta, Stefano Rausei, Aleix Martinez-Perez, Giovanni Bellanova, Vinicius Cordeiro Fonseca, Fernando Hernández, Athanasios Marinis, Wellington Fernandes, Martha Quiodettis, Miklosh Bala, Andras Vereczkei, Rafael L. Curado, Gustavo Pereira Fraga, Bruno M. Pereira, Mahir Gachabayov, Guillermo Perez Chagerben, Miguel Leon Arellano, Sefa Ozyazici, Gianluca Costa, Tugan Tezcaner, Luca Ansaloni

**Affiliations:** 1 General, Emergency and Trauma Surgery Department, Papa Giovanni XXIII Hospital, Piazza OMS 1, 24127 Bergamo, Italy; 2 Emergency Surgery Department, Parma University Hospital, Parma, Italy; 3 Virginia Commonwealth University, Richmond, VA USA; 4 General Surgery, Letterkenny Hospital, Donegal, Ireland; 5 General and Emergency Surgery Department, Macerata Hospital, Macerata,, Italy; 6 Neuro Intensive Care Unit Department, Papa Giovanni XXIII Hospital, Bergamo, Italy; 7 Emergency Surgery Hospital, Bucharest, Romania; 8 Emergency Surgery Department, Ferrara University Hospital, Ferrara, Italy; 9 Science Research of Emergency Care N. A., Djanelidze, Russia; 10 Azienda Ospedaliera Universitaria Pisana, Pisa, Italy; 11 Kipshidze Central University Hospital, Kipshidze, Georgia; 12 HPB Surgery, Fondazione IRCCS Cà Granda Ospedale Maggiore Policlinico, Milano, Italy; 13 University Hospital “Prof Stoian Kirkovich” AD, Stara Zagora, Bulgaria; 14 Fourth Surgical Department, Hospital George Papanikolau, Aristotle University, Thessaloniki, Greece; 15 General Surgery, Infermi Hospital, Rimini, Italy; 16 Pediatric Intensive Care Unit, Papa Giovanni XXIII Hospital, Bergamo, Italy; 17 LAS + USC Medical Centre, Los Angeles, California USA; 18 Pediatric Intensive Care Unit, Hannover University Hospital, Hannover, Germany; 19 Khuala Krai Hospital, Kuala Krai, Malaysia; 20 Mansoura Faculty of Medicine, Mansoura, Egypt; 21 Medical University of Plovdiv, Plovdiv, Bulgaria; 22 Soroka Medical Centre, Beersheba, Israel; 23 University Hospital of Trauma, Tirana, Albania; 24 Emergency and General Surgery, Fondazione IRCCS Cà Granda Ospedale Maggiore Policlinico, Milano, Italy; 25 Oxford University Hospital, Oxford, UK; 26 John Radcliffe Hospital, Oxford, UK; 27 Erzincan University Faculty of Medicine Mengucek Gazi Training Research Hospital Erzincan, Erzincan, Turkey; 28 Chang Gung Memorial Hospital, Taoyuan, Taiwan; 29 Ospedale Maggiore, Lodi, Italy; 30 Ospedale di Circolo e Fondazione Macchi, University of Insubria, Varese, Italy; 31 Hospital Universitario Doctor Peset, Valencia, Spain; 32 S.S. Annunziata Hospital, Taranto, Italy; 33 Hospital Santa Virgínia, São Paulo, Brazil; 34 Hospital Central Militar, Mexico City, Mexico; 35 Tzaneio General Hospital of Piraeus, Piraeus, Greece; 36 Hospital Regional de Sao Jose, San Jose, Brazil; 37 Hospital Santo Tomás, Panama City, Panama; 38 Hadassah Hebrew University Medical Center, Jerusalem, Israel; 39 Department of Surgery, Medical School University Pécs, Pécs, Hungary; 40 Hospital De Clinicas Da Unicamp, Campinas, Brazil; 41 Vladimir City Clinical Hospital of Emergency Medicine, Vladimir City, Russia; 42 University Hospital, Cuenca, Ecuador; 43 Hospital La Paz, Madrid, Spain; 44 Adana Numune Training and Research Hospital, Department of Surgery, Adana, Turkey; 45 Ospedale Sant’ Andrea University Hospital Sapienza, Rome, Italy; 46 Baskent University School of Medicine, Ankara, Turkey

## Erratum

The original article [1] mistakenly incorporated a sentence within the Abstract that stated:

“Mean BMI: 36 +/- 5.6”

This statement was incorrect and has now been removed from the original article.
